# A nonlinear rotation-free shell formulation with prestressing for vascular biomechanics

**DOI:** 10.1038/s41598-020-74277-5

**Published:** 2020-10-16

**Authors:** Nitesh Nama, Miquel Aguirre, Jay D. Humphrey, C. Alberto Figueroa

**Affiliations:** 1 Department of Surgery, University of Michigan, Ann Arbor, MI USA; 2 Mines Saint-Étienne, Univ Lyon, Univ Jean Monnet, INSERM, U 1059 Sainbiose, Centre CIS, 42023 Saint-Étienne, France; 3 Department of Biomedical Engineering, Yale University, New Haven, CT USA; 4 Department of Biomedical Engineering, University of Michigan, Ann Arbor, MI USA

**Keywords:** Biomedical engineering, Applied mathematics

## Abstract

We implement a nonlinear rotation-free shell formulation capable of handling large deformations for applications in vascular biomechanics. The formulation employs a previously reported shell element that calculates both the membrane and bending behavior via displacement degrees of freedom for a triangular element. The thickness stretch is statically condensed to enforce vessel wall incompressibility via a plane stress condition. Consequently, the formulation allows incorporation of appropriate 3D constitutive material models. We also incorporate external tissue support conditions to model the effect of surrounding tissue. We present theoretical and variational details of the formulation and verify our implementation against axisymmetric results and literature data. We also adapt a previously reported prestress methodology to identify the unloaded configuration corresponding to the medically imaged in vivo vessel geometry. We verify the prestress methodology in an idealized bifurcation model and demonstrate the significance of including prestress. Lastly, we demonstrate the robustness of our formulation via its application to mouse-specific models of arterial mechanics using an experimentally informed four-fiber constitutive model.

## Introduction

Computational approaches to simulate cardiovascular mechanics in patient-specific anatomies have attracted significant interest owing to their applications in disease research, medical device design, and surgical planning^1–5^. To model blood flow in compliant arteries, various fluid-structure interaction (FSI) techniques including Arbitrary Lagrangian-Eulerian and immersed methods have emerged^6–8^. Modeling both the fluid and solid domain using three-dimensional elements has resulted in high computational costs for complex, patient-specific geometries, posing a significant challenge to achieve results in a clinically relevant time-frame, thus limiting broad adoption in clinical practices. The computational cost of these methods can be greatly reduced by recognizing that most arteries and veins are characterized by a low wall thickness to radius ratio and can therefore be modeled as a thin structure, an assumption supported further by the existence of residual stresses that tend to homogenize the stress distribution across the wall. The Coupled Momentum Method (CMM)^9^ utilizes this simplification by employing a 2D representation of the vessel wall for modeling blood flow in compliant arteries. Specifically, the CMM employs a linear membrane formulation, resulting in minimal additional computational costs compared to methods with rigid wall approximation. The CMM has been implemented within open-source cardiovascular modeling environments, SimVascular^10^ and CRIMSON^11^, and has been utilized in numerous cardiovascular studies^12–15^. Nevertheless, the CMM is limited due to the use of fixed computational grids and the assumption of geometric and material linearity for the vessel wall behavior. To better capture large deformations and fully nonlinear anisotropic material behaviors, such as in highly compliant regions (e.g., ascending thoracic aorta), the CMM needs to be extended to a fully nonlinear formulation that accounts for both the geometric and material nonlinearities of the vessel wall.

The first essential step towards such a nonlinear FSI framework is development of a robust and computationally efficient nonlinear solid mechanics formulation for the vessel wall. A direct extension of the CMM solid kinematics to a nonlinear membrane model represents the simplest and most computationally efficient option. Yet, a nonlinear membrane model is limited due to the lack of bending stiffness, thereby rendering it inapplicable in regions of high curvature such as branches and bifurcations. Therefore, a nonlinear shell formulation that is capable of handling large deformations, including both membrane and bending modes, is preferred for modeling complex, patient-specific anatomies.

The Kirchhoff-Love theory, which assumes negligible transverse shear deformations, applies to thin pressurized structures characterized by a low thickness to radius ratio. However, given the widespread use of linear elements in solid mechanics, the concomitant necessity of $C^1$ continuity represents a major obstacle in the development of efficient finite element implementations^16^. Consequently, thick shell formulations based on Reissner-Mindlin theory requiring only $C^0$ continuity have been extensively used (see reference^17^ for a comprehensive discussion). Such formulations exhibit various locking phenomena (including shear locking), however, and are computationally expensive due to the large number of degrees of freedom necessary to obtain thin wall solutions with reasonable precision^18^ (e.g., linear triangular shell elements with 54 degrees of freedom per element^19^). We refer the reader to references^20–23^ for excellent reviews of shell formulations. Recently, various isogeometric shell models with inherent high continuity in basis functions have been proposed to satisfy the $C^1$ continuity requirement in Kirchhoff-Love theory for thin shells^16,24–26^. Another promising approach is to express the curvature field in terms of the displacement of nodes of neighboring elements, thereby avoiding the need for rotational degrees of freedom^18,27–29^. Numerous rotation-free shell elements have been reported, including the basic shell triangle (BST) element^27,28^, curvature calculation through enhanced geometry (CEG) shell element^18^, and subdivision surfaces^29^.

The current work aims to implement a nonlinear structural formulation for realizing our overall vision of developing a strongly coupled, monolithic, computationally efficient, nonlinear FSI framework for vascular biomechanics. The lack of rotational degrees of freedom and the ability to handle non-structured meshes for complex, subject-specific geometries is crucial for such an FSI framework. A rotation-free nonlinear shell formulation for vascular biomechanics will allow vessel wall nonlinearities to be incorporated in biomechanical analyses of complex subject-specific geometries and future extensions to FSI problems via an adaption of the CMM to large displacements.

Another important issue in vascular biomechanics is the lack of knowledge of the stress-free configuration in vivo. Specifically, models constructed from in vivo medical imaging necessarily represent both residually stressed (resulting from vascular development) and pre-stressed (e.g. due to axial preloads and in vivo pressures) vessels. To address this issue, various methods have been proposed either to estimate the state of stress in the in vivo geometry under in vivo loading^30–33^ or to identify the stress-free configuration directly^34–36^. One such method is a fixed-point iterative approach introduced by Sellier for general elastostatics problems^37^ and by Bols et al.^38^ for cardiovascular biomechanics. This method has been widely used in biomechanical modeling due to its algorithmic simplicity, capability to handle arbitrary material models, and ease of coupling with any finite element package that allows access to nodal displacements^39–41^.

In this work, we meld two well-known approaches. First, we adapt and expand the rotation-free triangular shell element reported by Oñate and Flores^27,28^, employing linear triangular shell elements where both the membrane and curvature fields are approximated in terms of the displacement of an element patch comprising the chosen element and three adjacent elements. We perform static condensation of the thickness stretch via incompressibility and a plane stress condition to allow 3D constitutive material models. We also incorporate external tissue support conditions to model the effect of perivascular tethering. Second, we adapt Sellier’s prestress approach for a shell formulation to determine the unloaded traction-free configurations associated with medically-obtained, subject-specific in vivo vessel geometries. Toward this end, we describe the theoretical and implementation details of the proposed shell formulation and verify it against axisymmetric results and literature data. We also verify the prestress methodology in an idealized bifurcation model and demonstrate its significance in predicting arterial wall deformations. Lastly, we demonstrate the utility and robustness of our melded formulation via its application to subject-specific vascular mechanics in the mouse, using a constitutive equation for the wall that has been validated against biaxial mechanical data for murine arteries.

## Theoretical formulation

### Notation

We use lowercase and uppercase symbols to define quantities in the current and reference (material) configuration, respectively. Symbols with Greek subscripts/superscripts denote in-plane quantities where indices take values $\{1,2\}$ while symbols with Latin subscripts/superscripts denote quantities corresponding to 3D and indices take values $\{1,2,3\}$. We follow Einstein’s index notation where a repeated symbol denotes a summation unless stated otherwise.

### Differential geometry of shell

**Figure 1:**
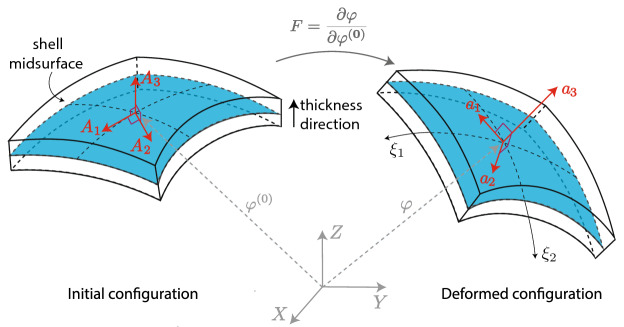
Schematic drawing illustrating the basis vectors in the initial and deformed configuration.

Using the Kirchhoff hypothesis of straight and normal cross-sections, the shell continuum can be described by the midsurface position and the associated normal vector field. Given the position ${\varvec{\varphi }}^{(0)}$ of a point on the midsurface of the shell in the initial configuration, the associated tangent basis vectors are defined as

1 $$\begin{aligned} {\mathbf{B }}_\alpha =\frac{\partial {\varvec{\varphi }}^{(0)}}{\partial \xi _\alpha }, \quad {\mathbf{B }}_3=\frac{{\mathbf{B }}_1\times {\mathbf{B }}_2}{\Vert {\mathbf{B }}_1\times {\mathbf{B }}_2\Vert }, \end{aligned}$$

where $\xi _1$ and $\xi _2$ are the surface convective coordinates used to parametrize the shell midsurface. Following Gruttman and Taylor^42^, an orthonormal basis is defined in the initial configuration as (see Fig. 1)

2 $$\begin{aligned} {\mathbf{A }}_3=\frac{{\mathbf{B }}_1\times {\mathbf{B }}_2}{\Vert {\mathbf{B }}_1 \times {\mathbf{B }}_2\Vert },\quad {\mathbf{A }}_1=\frac{{\mathbf{B }}_1}{\Vert {\mathbf{B }}_1\Vert },\quad {\mathbf{A }}_2={\mathbf{A }}_3\times {\mathbf{A }}_1, \end{aligned}$$

with associated coordinates $s_\alpha$ indirectly defined as

3 $$\begin{aligned} {\mathbf{A }}_\alpha = \frac{\partial {\varvec{\varphi }}^{(0)}}{\partial s_\alpha }. \end{aligned}$$

Similarly, the basis vectors at a position ${\varvec{\varphi }}$ of a point on the midsurface of the shell in the current configuration can be defined as

4 $$\begin{aligned} {\mathbf{a }}_\alpha = \frac{\partial {\varvec{\varphi }}}{\partial s_\alpha }. \end{aligned}$$

Using these definitions and the Kirchhoff hypothesis, positions $\mathbf{X }$ and $\mathbf{x }$ of a general point in the initial and current configuration, respectively, of the shell continuum can be described as a function of the midsurface position and associated unit normal vector as

5 $$\begin{aligned} \mathbf{X }(\xi _1, \xi _2, \xi _3)&= {\varvec{\varphi }}^{(0)}(\xi _1, \xi _2) + \xi _3 {\mathbf{A }}_3 (\xi _1, \xi _2), \end{aligned}$$

6 $$\begin{aligned} \mathbf{x }(\xi _1, \xi _2, \xi _3)&= {\varvec{\varphi }}(\xi _1, \xi _2) + \xi _3 {\mathbf{a }}_3 (\xi _1, \xi _2), \end{aligned}$$

where $\xi _3 (-\frac{H}{2} \le \xi _3 \le \frac{H}{2})$ denotes the perpendicular distance of the point to the midsurface with $H$ being the shell thickness in the reference configuration.

The covariant basis vectors at a general point in the initial configuration of the shell continuum are given as ${\mathbf{G }}_\alpha =\frac{\partial \mathbf{X }}{\partial s_\alpha }$ and can be expressed in terms of midsurface basis vectors as:

7 $$\begin{aligned} {\mathbf{G }}_\alpha&= {\mathbf{A }}_\alpha + \xi _3 {\mathbf{A }}_{3,\alpha }, \end{aligned}$$

8 $$\begin{aligned} {\mathbf{G }}_3&= {\mathbf{A }}_3, \end{aligned}$$

where $(\mathbf {\cdot })_{,\alpha }$ denotes the partial derivative of a quantity with respect to $s_\alpha$. The contravariant vectors can be obtained as

9 $$\begin{aligned} {\mathbf{G }}^\alpha&= {G}^{\alpha \beta } {\mathbf{G }}_\beta , \qquad {G}^{\alpha \gamma } {G}_{\gamma \beta }=\delta ^{\alpha }_{\,\beta }, \qquad {G}_{\alpha \beta }={\mathbf{G }}_\alpha \cdot {\mathbf{G }}_\beta . \end{aligned}$$

Therefore, the metrics can be obtained as

10 $$\begin{aligned} {G}_{\alpha \beta }&= {A}_{\alpha \beta } +2 \xi _3 \kappa ^{(0)}_{\alpha \beta } +\xi _3^2 {\mathbf{A }}_{3,\alpha } \cdot {\mathbf{A }}_{3,\beta }, \end{aligned}$$

11 $$\begin{aligned} {G}_{\alpha 3}&= {G}_{3 \alpha } ={\mathbf{A }}_\alpha \cdot {\mathbf{A }}_3 +\xi _3 {\mathbf{A }}_{3,\alpha } \cdot {\mathbf{A }}_3=0, \end{aligned}$$

12 $$\begin{aligned} {G}_{33}&={A}_{33}=1, \end{aligned}$$

where ${A}_{\alpha \beta }$ are the components of the covariant metrics (first fundamental form) in the initial configuration given as

13 $$\begin{aligned} {A}_{\alpha \beta } = {\mathbf{A }}_\alpha \cdot {\mathbf{A }}_\beta , \end{aligned}$$

and $\kappa ^{(0)}_{\alpha \beta }$ are the components of the curvature (second fundamental form) of the midsurface in the initial configuration given as

14 $$\begin{aligned} \kappa ^{(0)}_{\alpha \beta } = \frac{1}{2} ({\mathbf{A }}_\alpha \cdot {\mathbf{A }}_{3,\beta } + {\mathbf{A }}_\beta \cdot {\mathbf{A }}_{3,\alpha }). \end{aligned}$$

We linearize strain through the thickness and therefore neglect the quadratic term in Eq. (10) to obtain^16^:

15 $$\begin{aligned} {G}_{\alpha \beta }&= {A}_{\alpha \beta } +2 \xi _3 \kappa ^{(0)}_{\alpha \beta }. \end{aligned}$$

Similarly, using the basis vectors in the current configuration ${\mathbf{g }}_\alpha =\frac{\partial \mathbf{x }}{\partial s_\alpha }$, the metrics in the current configuration are

16 $$\begin{aligned} {g}_{\alpha \beta }&= {a}_{\alpha \beta } +2 \xi _3 \kappa _{\alpha \beta }, \end{aligned}$$

with

17 $$\begin{aligned} {a}_{\alpha \beta } = {\mathbf{a }}_\alpha \cdot {\mathbf{a }}_\beta , \qquad \qquad \kappa _{\alpha \beta } = \frac{1}{2} ({\mathbf{a }}_\alpha \cdot {\mathbf{a }}_{3,\beta } + {\mathbf{a }}_\beta \cdot {\mathbf{a }}_{3,\alpha }). \end{aligned}$$

This expression shows that ${g}_{\alpha \beta }$ are not components of the unit tensor in the initial configuration for curved surfaces (i.e. where $\kappa ^{(0)}_{\alpha \beta }\ne 0$). Therefore, following Flores and Oñate^27^, we approximate the covariant components

18 $$\begin{aligned} {g}_{\alpha \beta } = {a}_{\alpha \beta } +2 \xi _3 \chi _{\alpha \beta }, \end{aligned}$$

where $\chi _{\alpha \beta }$ denotes the change in curvature of the midsurface and is

19 $$\begin{aligned} \chi _{\alpha \beta }=\kappa _{\alpha \beta }-\kappa ^{(0)}_{\alpha \beta }. \end{aligned}$$

Note that the orthonormal basis equation (2) is defined only in the initial configuration and the corresponding basis in the deformed configuration is, in general, not orthonormal (see Fig. 1). Furthermore, the above equations do not reflect changes in thickness during the deformation, which will be considered later.

### Shell kinematics

Using the basis vectors defined in Eqs. (7)–(9), the deformation gradient tensor can be written

20 $$\begin{aligned} {\mathbf{F }}=\frac{\partial \mathbf{x }}{\partial \mathbf{X }} =\frac{\partial \mathbf{x }}{\partial s_\alpha }\otimes \frac{\partial s_\alpha }{\partial \mathbf{X }} ={\mathbf{g }}_i\otimes {\mathbf{G }}^i, \end{aligned}$$

and the right Cauchy-Green tensor as

21 $$\begin{aligned} {\mathbf{C }}={\mathbf{F }}^T {\mathbf{F }}={g}_{ij} {\mathbf{G }}^i \otimes {\mathbf{G }}^j. \end{aligned}$$

Here, the transverse components ${g}_{\alpha 3}={g}_{3\alpha } = 0$ since ${\mathbf{g }}_\alpha \cdot {\mathbf{g }}_3 = 0$. Furthermore, Eq. (21) indicates that the covariant coefficients of the right Cauchy-Green tensor and the metrics in the deformed configuration are identical. However, since ${g}_{33}={\mathbf{a }}_3 \cdot {\mathbf{a }}_3 = 1$, this equation does not reflect the change in shell thickness. Consequently, to account for the thickness deformation, Eq. (21) needs to be replaced by^16^

22 $$\begin{aligned} {\mathbf{C }}={C}_{ij} {\mathbf{G }}^i \otimes {\mathbf{G }}^j, \end{aligned}$$

with

23 $$\begin{aligned} {C}_{ij}= \begin{bmatrix} {g}_{11} &{} {g}_{12} &{} 0\\ {g}_{21} &{} {g}_{22} &{} 0\\ 0 &{} 0 &{} {C}_{33} \end{bmatrix}, \end{aligned}$$

where ${C}_{33}$ will be obtained via plane stress and incompressiblity conditions, as described in “Constitutive models” section. This allows the Jacobian determinant to be expressed as

24 $$\begin{aligned} {J}=\sqrt{\text {det}({\mathbf{C }})}={J}_0\sqrt{{C}_{33}}, \end{aligned}$$

where ${J}_0=\sqrt{\frac{| {g}_{\alpha \beta }|}{| {G}_{\alpha \beta }|}}$ denotes the in-plane Jacobian determinant. Equation (24), in combination with the incompressibility condition

25 $$\begin{aligned} {J}=1, \end{aligned}$$

yields

26 $$\begin{aligned} {C}_{33}={J}_0^{-2}. \end{aligned}$$

This coupling of out-of-plane components with the in-plane metrics ensures satisfaction of the incompressibility constraint.

Next, the Green-Lagrange strain tensor can be written

27 $$\begin{aligned} {\mathbf{E }}={E}_{ij} {\mathbf{G }}^i \otimes {\mathbf{G }}^j, \end{aligned}$$

with

28 $$\begin{aligned} {E}_{ij}=\frac{1}{2}({C}_{ij}-{G}_{ij}). \end{aligned}$$

Here, the transverse shear strains vanish since ${C}_{\alpha 3}={G}_{\alpha 3}=0$. Furthermore, the transverse normal strain ${E}_{33}$ will be statically condensed by employing plane stress and incompressibility constraint, as described in “Constitutive models” section^16^. Therefore, only the in-plane components remain to be considered and are given as

29 $$\begin{aligned} {E}_{\alpha \beta }= \frac{1}{2}({C}_{\alpha \beta }-{G}_{\alpha \beta })={E}^\text {m}_{\alpha \beta } + \xi _3 \chi _{\alpha \beta }, \end{aligned}$$

where ${E}^\text {m}_{\alpha \beta }$ denotes the components of the membrane contribution to the Green-Lagrange strain tensor

30 $$\begin{aligned} {E}^\text {m}_{\alpha \beta }=\frac{1}{2}\big [({a}_{\alpha \beta }-{A}_{\alpha \beta }) \big ], \end{aligned}$$

and $\chi _{\alpha \beta }$ denotes the change in curvature of the midsurface given by Eq. (19).

### Constitutive models

Next, consider constitutive equations for the shell formulation from a general 3D continuum constitutive model characterized by an elastic strain energy function $\psi _\text {el}({\mathbf{C }})$. To enforce incompressibility, the elastic strain energy function $\psi _\text {el}({\mathbf{C }})$ is augmented with a constraint term using a Lagrange multiplier $p$ as

31 $$\begin{aligned} \psi = -p({J}-1) + \psi _\text {el}({\mathbf{C }}). \end{aligned}$$

For the shell model, the additional unknown $p$ can be determined using a plane stress condition^16^. Following Kiendl et al.^16^, we first derive the 3D components of the second Piola-Kirchhoff stress tensor (${\mathbf{S }}={S}^{ij} {\mathbf{G }}_i \otimes {\mathbf{G }}_j$) while considering $p$ to be a function of ${C}_{ij}$ as

32 $$\begin{aligned} {S}^{ij} = 2\frac{\partial \psi }{\partial {C}_{ij}} = 2\frac{\partial \psi _\text {el}}{\partial {C}_{ij}}-2\frac{\partial p}{\partial {C}_{ij}}({J}-1)-2p\frac{\partial {J}}{\partial {C}_{ij}}. \end{aligned}$$

Correspondingly, the components of the stiffness tensor ${\mathcal {\mathbf{C}}}={\mathcal {C}}^{ijkl} {\mathbf{G }}_i \otimes {\mathbf{G }}_j \otimes {\mathbf{G }}_k \otimes {\mathbf{G }}_l$ are

33 $$\begin{aligned} {\mathcal {C}}^{ijkl}&=4\frac{\partial ^2 \psi }{\partial {C}_{ij} \partial {C}_{kl}} \nonumber \\ &=4\frac{\partial ^2 \psi _\text {el}}{\partial {C}_{ij} \partial {C}_{kl}}-4\frac{\partial ^2 p}{\partial {C}_{ij} \partial {C}_{kl}}({J}-1)-4\frac{\partial p}{\partial {C}_{ij}}\frac{\partial {J}}{\partial {C}_{kl}} \\ & \quad -4\frac{\partial {J}}{\partial {C}_{ij}}\frac{\partial p}{\partial {C}_{kl}}-4\frac{\partial ^2 {J}}{\partial {C}_{ij} \partial {C}_{kl}}, \end{aligned}$$

with derivatives of the Jacobian determinant given as

34 $$\begin{aligned} \frac{\partial {J}}{ \partial {C}_{ij}}&=\frac{1}{2}{J}\check{{C}}^{ij}, \end{aligned}$$

35 $$\begin{aligned} \frac{\partial ^2 {J}}{\partial {C}_{ij} \partial {C}_{kl}}&=\frac{1}{4}{J}\big ( \check{{C}}^{ij} \check{{C}}^{kl}-\check{{C}}^{ik}\check{{C}}^{jl} -\check{{C}}^{il}\check{{C}}^{jk} \big ), \end{aligned}$$

where $\check{{C}}^{ij}$ denote the components of the inverse of the Cauchy-Green tensor. Let the term ‘plane stress’ refer to the state of zero normal transverse stress. Using the plane stress condition

36 $$\begin{aligned} {S}^{33}=0, \end{aligned}$$

with Eqs. (24), (25), (26), (32), and (34), we obtain

37 $$\begin{aligned} p=2\frac{\partial \psi _\text {el}}{\partial {C}_{33}}{C}_{33}. \end{aligned}$$

The corresponding derivative is

38 $$\begin{aligned} \frac{\partial p}{\partial {C}_{ij}}=2\bigg ( \frac{\partial ^2 \psi _\text {el}}{\partial {C}_{33} \partial {C}_{ij}} {C}_{33}+\frac{\partial \psi _\text {el}}{\partial {C}_{33}} \delta ^{i3} \delta ^{j3} \bigg ), \end{aligned}$$

where $\delta ^{ij}$ denotes the Kronecker delta. Substituting these relations into Eqs. (32) and (33) and using Eqs. (25), (34), and (35), we obtain

39 $$\begin{aligned} {S}^{ij}&= 2\frac{\partial \psi _\text {el}}{\partial {C}_{ij}}-2 \frac{\partial \psi _\text {el}}{\partial {C}_{33}}{C}_{33} \check{{C}}^{ij}, \end{aligned}$$

40 $$\begin{aligned} {\mathcal {C}}^{ijkl}&=4\frac{\partial ^2 \psi _\text {el}}{\partial {C}_{ij} \partial {C}_{kl}} -4\bigg ( \frac{\partial ^2 \psi _\text {el}}{\partial {C}_{33} \partial {C}_{ij}} {C}_{33}+\frac{\partial \psi _\text {el}}{\partial {C}_{33}} \delta ^{i3} \delta ^{j3} \bigg ) \check{{C}}^{kl} -4 \check{{C}}^{ij}\bigg ( \frac{\partial ^2 \psi _\text {el}}{\partial {C}_{33} \partial {C}_{kl}} {C}_{33}+\frac{\partial \psi _\text {el}}{\partial {C}_{33}} \delta ^{k3} \delta ^{l3} \bigg ) \nonumber \\ & \quad -2 \frac{\partial \psi _\text {el}}{\partial {C}_{33}}{C}_{33} \big ( \check{{C}}^{ij} \check{{C}}^{kl}-\check{{C}}^{ik}\check{{C}}^{jl}-\check{{C}}^{il} \check{{C}}^{jk} \big ). \end{aligned}$$

These expressions define the 3D stress and elasticity tensor for a general incompressible material under plane stress. To express the in-plane components of these tensors needed for the shell formulation, we perform static condensation of the transverse normal strain so that the entire shell kinematics can be described completely by midsurface quantities. Specifically, the plane stress condition in Eq. (36) is used to eliminate the transverse normal strain ${E}_{33}$ as

41 $$\begin{aligned} {S}^{33}={\mathcal {C}}^{33\alpha \beta }{E}_{\alpha \beta } + {\mathcal {C}}^{3333}{E}_{33}=0 \implies {E}_{33}=-\frac{{\mathcal {C}}^{33\alpha \beta }}{{\mathcal {C}}^{3333}}{E}_{\alpha \beta }. \end{aligned}$$

Therefore, coefficients of the statically condensed stiffness tensor $\breve{{\mathcal {C}}}^{\alpha \beta \gamma \delta }$ are^16^

42 $$\begin{aligned} \breve{{\mathcal {C}}}^{\alpha \beta \gamma \delta } = {\mathcal {C}}^{\alpha \beta \gamma \delta }-\frac{{\mathcal {C}}^{\alpha \beta 33} {\mathcal {C}}^{33\gamma \delta }}{{\mathcal {C}}^{3333}}. \end{aligned}$$

Substituting $\check{{C}}^{\alpha \beta }={g}^{\alpha \beta }$ and ${C}_{33}={J}_0^{-2}$ in Eqs. (39) and (40) and using Eq. (42), the in-plane components of stress and stiffness tensor needed for the shell formulation are

43 $$\begin{aligned} {S}^{\alpha \beta }&= 2\frac{\partial \psi _\text {el}}{\partial {C}_{\alpha \beta }}-2 \frac{\partial \psi _\text {el}}{\partial {C}_{33}} {J}_0^{-2} {g}^{\alpha \beta }, \end{aligned}$$

44 $$\begin{aligned} \breve{{\mathcal {C}}}^{\alpha \beta \gamma \delta }&=4\frac{\partial ^2 \psi _\text {el}}{\partial {C}_{\alpha \beta } \partial {C}_{\gamma \delta }} +4 \frac{\partial ^2 \psi _\text {el}}{\partial {C}_{33}^2} {J}_0^{-4} {g}^{\alpha \beta } {g}^{\gamma \delta } -4 \frac{\partial ^2 \psi _\text {el}}{\partial {C}_{33} \partial {C}_{\alpha \beta }} {J}_0^{-2} {g}^{\gamma \delta } -4 \frac{\partial ^2 \psi _\text {el}}{\partial {C}_{33} \partial {C}_{\gamma \delta }} {J}_0^{-2} {g}^{\alpha \beta } \nonumber \\ & \quad +2 \frac{\partial \psi _\text {el}}{\partial {C}_{33}} {J}_0^{-2}\big ( 2 {g}^{\alpha \beta } {g}^{\gamma \delta } +{g}^{\alpha \gamma } {g}^{\beta \delta } +{g}^{\alpha \delta } {g}^{\beta \gamma }\big ) . \end{aligned}$$

Using these expressions, a through-thickness integration yields the stress resultants

45 $$\begin{aligned} N^{\alpha \beta }&=\int _{-H/2}^{H/2} {S}^{\alpha \beta } \,d\xi _3, \end{aligned}$$

46 $$\begin{aligned} M^{\alpha \beta }&=\int _{-H/2}^{H/2} {S}^{\alpha \beta } \xi _3 \,d\xi _3. \end{aligned}$$

Equations (43) and (44) provide a consistent method of obtaining in-plane components of stress and stiffness from arbitrary 3D constitutive models. In this work, we consider two different constitutive models:

#### Mooney-Rivlin

The elastic strain energy for Mooney-Rivlin model is

47 $$\begin{aligned} \psi _\text {el}= c_1(I_C-3) + c_2(II_C- 3), \end{aligned}$$

where $c_1$, $c_2$ are the material parameters and $I_C$, $II_C$ are the first and second principal invariants of right Cauchy-Green tensor given as

48 $$\begin{aligned} I_C&= \text {tr}({\mathbf{C }}), \end{aligned}$$

49 $$\begin{aligned} II_C&=\frac{1}{2} \big [\text {tr}({\mathbf{C }})^2 -\text {tr}({\mathbf{C }}^2)\big ]. \end{aligned}$$

For the special case of $c_2=0$, this model reduces to the incompressible Neo-Hookean model.

#### Four-fiber family model

The four-fiber family model characterizes arterial wall as a composite structure comprising an elastin-dominated isotropic matrix and four families of embedded collagen fibers. The elastic strain energy is^43–45^

50 $$\begin{aligned} \psi _\text {el}= \frac{c}{2} (I_C-3) +\sum _{k=1}^4 \frac{c_1^k}{4 c_2^k} \Big [ e^{c_2^k [(\lambda ^k)^2-1]^2}-1 \Big ], \end{aligned}$$

where $c$, $c_1^k$, $c_2^k$ are material parameters, $I_C$ is again the first invariant of right Cauchy-Green tensor and $\lambda ^k=\sqrt{\mathbf{M }^k \cdot {\mathbf{C }}\mathbf{M }^k}$ is the stretch experienced by the *k*-th fiber family that is oriented along the direction $\mathbf{M }^k = [0,\sin \alpha _0^k, \cos \alpha _0^k]^T$ in the reference configuration. The four-fiber families are considered to be aligned along $\alpha _0^1=0^{\circ }$ (axial family), $\alpha _0^2=90^{\circ }$ (circumferential family) and $\alpha _0^{3,4}=\pm \alpha _0$ (symmetric diagonal families), where $\alpha _0$ is a free parameter. The four-fiber family model has recently been recommended as the first choice constitutive model to simulate arterial deformations that lie outside the range of experimental tests^46^ and has been extensively employed to describe the behavior of both human and murine arterial tissue^44,45^.

## Implementation

The weak form of linear momentum balance can be expressed as

51 $$\begin{aligned} \delta W^\text {kin}+\delta W^\text {int}-\delta W^\text {ext}=0, \end{aligned}$$

with

52 $$\begin{aligned} \delta W^\text {kin}&= \int _A\rho _0\ddot{\mathbf{u }} \cdot \delta \mathbf{u }H\,dA, \end{aligned}$$

53 $$\begin{aligned} \delta W^\text {int}&= \int _A[\mathbf{N }: \delta {\mathbf{E }}^\text {m}+ \mathbf{M }: \delta {\varvec{\chi }}] H\,dA, \end{aligned}$$

54 $$\begin{aligned} \delta W^\text {ext}&= \int _A\mathbf{f }\cdot \delta \mathbf{u }H\, dA, \end{aligned}$$

where $\rho _0$ is the mass density, $\ddot{\mathbf{u }}=\frac{\partial ^2 \mathbf{u }}{\partial t^2}$ the acceleration, $\mathbf{f }$ the external force, $\delta \mathbf{u }$ the virtual displacement, $\delta {\mathbf{E }}^\text {m}$ the variation of Green-Lagrange strain tensor, $\delta {\varvec{\chi }}$ the variation of the curvature, and $\mathbf{N }$, $\mathbf{M }$ are the force and bending stress resultants with their components given by Eqs. (45) and (46).

### Interpolation functions

**Figure 2:**
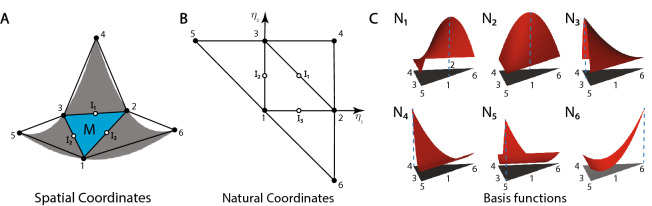
Schematic of the element patch comprising a master element and three adjacent elements in (**A**) spatial coordinates and (**B**) natural coordinates. (**C**) Plots of the six quadratic shape functions defined over the element patch.

Here, we closely follow Flores and Oñate^27^. Consider a surface discretization of the shell midsurface using a triangular mesh with linear shape functions associated with displacement degrees of freedom at each node. Since a linear triangular element is *flat*, the linear shape functions over an element are inadequate for the calculation of curvature. Therefore, following^27^, to calculate the membrane and bending behavior of a given element (referred as the master element), we employ an element patch comprising the given element and its three neighboring elements (see Fig. 2).

This element patch, comprising six nodes, allows us to consider a quadratic interpolation of the geometry that can be used to compute the membrane and curvature behavior. Figure 2A shows the current configuration of the element patch with the corresponding nodal positions of six nodes given in natural coordinates as (Fig. 2B)

$$\begin{aligned}&\text {node 1}:(\eta _1, \eta _2)=(0,0), \qquad\text {node 4}: (\eta _1, \eta _2)=(1,1),\\&\text {node 2}:(\eta _1, \eta _2)=(1,0), \qquad\text {node 5}: (\eta _1, \eta _2)=(-1,1),\\&\text {node 3}:(\eta _1, \eta _2)=(0,1), \qquad\text {node 6}: (\eta _1, \eta _2)=(1,-1), \end{aligned}$$

where the nodes of the neighboring elements are ordered such that nodes 4, 5, and 6 refer to those opposite nodes 1, 2, and 3, respectively. The standard linear shape functions defined on a three node triangle are

55 $$\begin{aligned} L_1=\eta _1, \qquad L_2=\eta _2, \qquad L_3=1-\eta _1-\eta _2. \end{aligned}$$

In addition, we define the following (quadratic) shape functions over the element patch as

$$\begin{aligned}&N^1=\eta _3+\eta _1\eta _2, \qquad&N^4=\frac{\eta _3}{2}(\eta _3-1),\\&N^2=\eta _1+\eta _2\eta _3, \qquad&N^5=\frac{\eta _1}{2}(\eta _1-1),\\&N^3=\eta _2+\eta _3\eta _1, \qquad&N^6=\frac{\eta _2}{2}(\eta _2-1), \end{aligned}$$

where $\eta _3=1-\eta _1-\eta _2$. Figure 2C shows these shape functions over the element patch, which allows us to approximate the position of shell midsurface as

56 $$\begin{aligned} {\varvec{\varphi }}= \sum _{a=1}^{6} N^a(\eta _1,\eta _2) {\varvec{\varphi }}^a. \end{aligned}$$

Additionally, we employ a single in-plane quadrature point per element. Similar to the EBST1 element reported by Oñate and Flores^28^, we employ an *assumed strain* approach where the gradients at the single quadrature point are approximated via the gradients calculated at the midpoints of the edges of the master element (denoted as $I_1$, $I_2$, and $I_3$ in Fig. 2). Referring to Fig. 2, the edges of the master element are numbered such that the edge *I* refers to the edge opposite to node *I* ($I=1,2,3$). It can be verified that this choice of shape functions ensures that the gradient at a given edge midpoint depends only on the four nodes surrounding the edge midpoint. Therefore, the gradient at an edge midpoint is unique for the two elements that share this edge since the same four nodes contribute to the gradient at edge midpoint, irrespective of the choice of element over which the gradient is calculated.

### Computation of membrane strain

The components of the membrane part of Green-Lagrange strain tensor in Eq. (30) are given in Voigt notation as

57 $$\begin{aligned} \begin{bmatrix} {E}^\text {m}_{11} \\ {E}^\text {m}_{22} \\ 2 {E}^\text {m}_{12} \end{bmatrix}&= \frac{1}{2}\begin{bmatrix} {a}_{11} -1 \\ {a}_{22}-1 \\ 2 {a}_{12} \end{bmatrix} . \end{aligned}$$

Using the assumed strain approach^28^, we calculate the components of metrics by averaging their values at the edge midpoints as

58 $$\begin{aligned} {a}_{\alpha \beta } = \frac{1}{3}\sum _{I=1}^3 {a}_{\alpha \beta }^I, \end{aligned}$$

where the superscript *I* indicates that the variable has been evaluated at the midpoint of the edge *I*. While a variety of other choices exist for approximating covariant metrics, the above approach satisfies the patch test and performs well for large deformations^47^. For the boundary edges (where one or more elements of the patch are missing), the gradient at the edge midpoint is taken to be equal to the gradient calculated over the master element as

59 $$\begin{aligned} {\varvec{\varphi }}_{,\alpha } \Big |_{\text {boundary edge}}={\varvec{\varphi }}_{,\alpha }^M= \sum ^{3}_{I=1} L^I_{,\alpha } {\varvec{\varphi }}^I, \end{aligned}$$

where the superscript $(\cdot )^M$ indicates that the quantity has been calculated using standard three-node linear shape functions defined in Eq. (55). Using Eqs. (4), (17) and (57), the variation of the membrane part of Green-Lagrange strain tensor is obtained as

60 $$\begin{aligned} \delta \begin{bmatrix} {E}^\text {m}_{11} \\ {E}^\text {m}_{22} \\ 2 {E}^\text {m}_{12} \end{bmatrix}&= \begin{bmatrix} {\varvec{\varphi }}_{,1} \cdot \delta {\varvec{\varphi }}_{,1} \\ {\varvec{\varphi }}_{,2} \cdot \delta {\varvec{\varphi }}_{,2} \\ {\varvec{\varphi }}_{,1} \cdot \delta {\varvec{\varphi }}_{,2} + {\varvec{\varphi }}_{,2} \cdot \delta {\varvec{\varphi }}_{,1} \end{bmatrix} . \end{aligned}$$

Further algebraic manipulations needed for implementation are in Supplementary Appendix B online.

### Computation of curvature

Following Oñate and Flores^28^, we assume the curvature to be uniform within each element which can be calculated as the mean over the master element given by

61 $$\begin{aligned} \kappa _{\alpha \beta }=-\frac{1}{A^M} \int _{A^M} {\mathbf{a }}_3 \cdot {\varvec{\varphi }}_{,\beta \alpha }\,dA^M, \end{aligned}$$

where $A^M$ is the area of the master element in the initial configuration. Integrating by parts and using the divergence theorem,

62 $$\begin{aligned} \kappa _{\alpha \beta }=-\frac{1}{A^M}\Bigg [\oint _{\Gamma ^M} {\mathbf{a }}_3 \cdot {\varvec{\varphi }}_{,\beta } n_{\alpha } \,d\Gamma ^M- \int _{A^M} ({\mathbf{a }}_3)_{,\alpha } \cdot {\varvec{\varphi }}_{,\beta } \,dA^M\Bigg ], \end{aligned}$$

where $n_{\alpha }$ denote components of outward normal to the element boundary. Similar to Oñate and Flores^28^, we consider the standard linear interpolation over the master element for the definition of midsurface normal ${\mathbf{a }}_3$ in the above expression, instead of the quadratic interpolation over the element patch to obtain

63 $$\begin{aligned} {\mathbf{a }}_3=\frac{{\varvec{\varphi }}_{,1}^M\times {\varvec{\varphi }}_{,2}^M}{| {\varvec{\varphi }}_{,1}^M\times {\varvec{\varphi }}_{,2}^M| }. \end{aligned}$$

This definition yields a uniform value of midsurface normal over the master element, resulting in zero contribution from the second term on the right hand side in Eq. (62). Therefore, the components of the curvature are

64 $$\begin{aligned} \begin{bmatrix} \kappa _{11} \\ \kappa _{22} \\ 2 \kappa _{12} \end{bmatrix} = -\frac{1}{A^M} \oint _{\Gamma ^M} \begin{bmatrix} n_1 &{} 0\\ 0 &{} n_2\\ n_2 &{} n_1 \end{bmatrix} \begin{bmatrix} {\varvec{\varphi }}_{,1} \cdot {\mathbf{a }}_3 \\ {\varvec{\varphi }}_{,2} \cdot {\mathbf{a }}_3 \end{bmatrix} \,d\Gamma ^M. \end{aligned}$$

Next, consider the line integral as the sum over the integration points (chosen as the edge midpoints) on the edges of the master element

65 $$\begin{aligned} \begin{bmatrix} \kappa _{11} \\ \kappa _{22} \\ 2 \kappa _{12} \end{bmatrix}&= -\frac{1}{A^M} \sum ^3_{I=1} l^I\begin{bmatrix} n_1 &{} 0\\ 0 &{} n_2\\ n_2 &{} n_1 \end{bmatrix}^I\begin{bmatrix} {\varvec{\varphi }}_{,1}^I\cdot {\mathbf{a }}_3 \\ {\varvec{\varphi }}_{,2}^I\cdot {\mathbf{a }}_3 \end{bmatrix} \nonumber \\&= 2 \sum ^3_{I=1} \begin{bmatrix} L^I_{,1} &{} 0\\ 0 &{} L^I_{,2}\\ L^I_{,2} &{} L^I_{,1} \end{bmatrix} \begin{bmatrix} {\varvec{\varphi }}_{,1}^I\cdot {\mathbf{a }}_3 \\ {\varvec{\varphi }}_{,2}^I\cdot {\mathbf{a }}_3 \end{bmatrix} , \end{aligned}$$

where $l^I$ denotes the length of $I$-th edge in the initial configuration. Equation (65) can be alternatively expressed as

66 $$\begin{aligned} \kappa _{\alpha \beta } = \mathbf{h }_{\alpha \beta } \cdot {\mathbf{a }}_3, \qquad \text {with} \qquad \mathbf{h }_{\alpha \beta }=\sum ^{3}_{I=1}(L^I_{,\alpha } {\varvec{\varphi }}^{I}_{,\beta }+L^I_{,\beta } {\varvec{\varphi }}^{I}_{,\alpha }), \end{aligned}$$

where the gradients ${\varvec{\varphi }}^{I}_{,\alpha }$ are calculated at the edge midpoints using quadratic interpolation. Equation (66) allows the curvatures to be interpreted as projections of vectors $\mathbf{h }_{\alpha \beta }$ over the normal vector. Therefore, if a different direction is chosen (e.g. the midsurface normal obtained via all six nodes of element patch), at an angle $\theta$ to the currently chosen uniform normal, this would have an influence of order $\theta ^2$ in the projection. This justifies the use of a normal defined solely on the master element, Eq. (63), to calculate the curvature.

We defer the treatment of the bending behavior of boundary edges to “Boundary conditions” section. Using Eqs. (19) and (66), the variation of the change in curvature can be expressed as

67 $$\begin{aligned} \delta \chi _{\alpha \beta } = \delta \kappa _{\alpha \beta } = \delta \mathbf{h }_{\alpha \beta } \cdot {\mathbf{a }}_3 + \mathbf{h }_{\alpha \beta } \cdot \delta {\mathbf{a }}_3, \end{aligned}$$

where the variations of $\mathbf{h }_{\alpha \beta }$ are obtained from Eq. (66) as

68 $$\begin{aligned} \delta \mathbf{h }_{\alpha \beta }=\sum ^{3}_{I=1}(L^I_{,\alpha } \delta {\varvec{\varphi }}^{I}_{,\beta }+L^I_{,\beta } \delta {\varvec{\varphi }}^{I}_{,\alpha }). \end{aligned}$$

For the second term in Eq. (67), the variations of ${\mathbf{a }}_3$ can be expressed as^27^

69 $$\begin{aligned} \delta {\mathbf{a }}_3 = \delta {a}_{31} \breve{\mathbf{a}}_{1} + \delta {a}_{32} \breve{\mathbf{a}}_{2}, \end{aligned}$$

where $\breve{\mathbf{a}}_{\alpha }$ are contravariant basis vectors in the master element defined using the linear interpolation and

70 $$\begin{aligned} \delta {a}_{31}&= -{\mathbf{a }}_3 \cdot \delta {\varvec{\varphi }}_{,1}^M, \end{aligned}$$

71 $$\begin{aligned} \delta {a}_{32}&= -{\mathbf{a }}_3 \cdot \delta {\varvec{\varphi }}_{,2}^M. \end{aligned}$$

Combining Eqs. (68)–(71) with Eq. (67) yields the final expression for the variation of curvature as seen in Supplementary Appendix B online.

### Boundary conditions

Treatment of boundary edges (with missing adjacent element) again follows Oñate and Flores^28^. For the sake of completeness, we mention the key expressions needed for the treatment of boundary conditions and refer the reader to reference^28^ for more details.

For the membrane terms, gradients at the boundary edges are considered to be equal those in the master element calculated using linear interpolation, see Eq. (59). However, for the curvature terms, $\mathbf{h }_{\alpha \beta }$ and $\delta \mathbf{h }_{\alpha \beta }$ are modified by considering a local-to-edge orthonormal basis $[{\varvec{\varphi }}_{,n}, {\bar{{\varvec{\varphi }}}}_{,q}]$ comprising the unit normal vector to the plane of symmetry ${\varvec{\varphi }}_{,n}$ and the unit normal vector along the boundary edge ${\bar{{\varvec{\varphi }}}}_{,q}$. This results, for a boundary edge defined by node *K* and *J*, in the following expressions for $\mathbf{h }_{\alpha \beta }$ and $\delta \mathbf{h }_{\alpha \beta }$ on the boundary edge midpoint:

72 $$\begin{aligned} \begin{bmatrix} \mathbf{h }^T_{11} \\ \mathbf{h }^T_{22} \\ 2 \mathbf{h }^T_{12} \end{bmatrix} ^{I}&= 2 \begin{bmatrix} L^I_{,1} &{} 0\\ 0 &{} L^I_{,2}\\ L^I_{,2} &{} L^I_{,1} \end{bmatrix} \begin{bmatrix} n_1 &{} -n_2 \\ n_2 &{} n_1 \end{bmatrix} \begin{bmatrix} {\varvec{\varphi }}_{,n}^T \\ {\varvec{\varphi }}_{,q}^T \end{bmatrix}, \end{aligned}$$

73 $$\begin{aligned} \delta \begin{bmatrix} \mathbf{h }^T_{11} \\ \mathbf{h }^T_{22} \\ 2 \mathbf{h }^T_{12} \end{bmatrix} ^{I}&= 2 \begin{bmatrix} L^I_{,1} &{} 0\\ 0 &{} L^I_{,2}\\ L^I_{,2} &{} L^I_{,1} \end{bmatrix} \begin{bmatrix} n_1 &{} -n_2 \\ n_2 &{} n_1 \end{bmatrix} \begin{bmatrix} {\mathbf {0}} \\ \frac{1}{L_0}(\delta \mathbf{u }^K- \delta \mathbf{u }^J)^T \end{bmatrix}, \end{aligned}$$

where the influence of change in the length of vector ${\varvec{\varphi }}_{,n}$ has been neglected.

### External tissue support

Various Robin boundary conditions have been proposed to model effects of the surrounding tissues on the vasculature^8,48^. These boundary conditions model the mechanical response of the external tissue via a lumped parameter model that relates the stress on the outer wall with its displacement and velocity. Using this approach, we consider an imposed traction on the shell surface given as

74 $$\begin{aligned} {\varvec{\sigma }}\cdot {\mathbf{a }}_3 = -k_s\mathbf{u }-k_d\mathbf{v }-p_0{\mathbf{a }}_3, \end{aligned}$$

where $k_s$ and $k_d$ are the coefficients associated with simplified elastic and the viscoelastic responses of the external tissue, respectively, and $p_0$ represents the extravascular pressure. In general, these parameters can depend both on space and time and can be tuned to match the available data on vessel wall displacement. We assume constant and uniform values, with $k_d=0$ for all cases.

### Time integration

Upon the spatial discretization of the weak form (51), a transient nonlinear system of equations is assembled and then discretized in time and solved using the Generalized-$\alpha$ method^49^ as given in Supplementary Appendix C online.

## Prestress methodology

To account for the in vivo state of stress in the medically imaged vessel geometry, we employ an iterative prestress method, first proposed by Sellier^37^. This method allows an easy interface with any standard finite element solid mechanics package and is applicable for arbitrary choice of material models^38,41^.

Sellier’s approach employs multiple forward simulations to iteratively update the nodal coordinates to identify the unloaded configuration. For 3D solid mesh elements, the unloaded geometry is described by the nodal coordinates of the mesh. In contrast, for a shell formulation, the unloaded geometry is described by nodal coordinates of shell midsurface and the corresponding thickness distribution over each mesh element. Therefore, the prestress approach for a shell formulation needs to iteratively update both the midsurface coordinates and the thickness distribution to identify the unloaded geometry.

**Figure 3:**
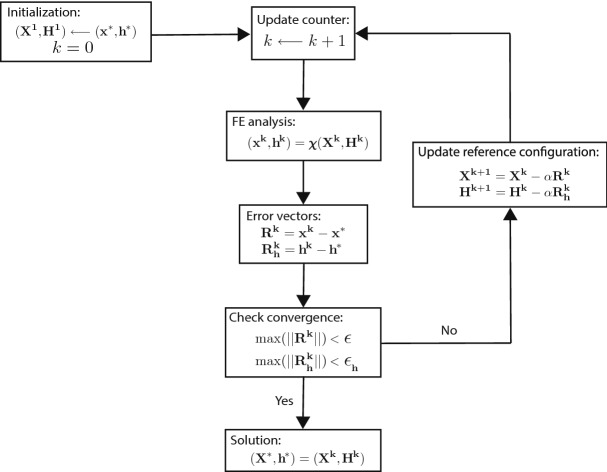
Algorithm for the prestress methodology to identify an appropriate traction-free reference configuration given available (in vivo) geometries from medical imaging.

The point of departure is the medically imaged vessel geometry, characterized by midsurface nodal coordinates and elemental thickness $({\mathbf {x}}^*,{\mathbf {h}}^*)$. Referring to Fig. 3, the medically imaged in vivo geometry is taken to be the first guess for the unloaded configuration, $({\mathbf {X}}^1,{\mathbf {H}}^1)$. Subsequently, the *k*-th iteration consists of applying the (known) in vivo loading to the *k*-th initial configuration to compute the *k*-th deformed configuration $({\mathbf {x}}^k,{\mathbf {h}}^k)$. At the end of each iteration, a nodal error vector is calculated as the difference between the coordinates of the deformed configuration and the measured in vivo configuration, ${\mathbf {R}}^k={\mathbf {x}}^k-{\mathbf {x}}^*$. In addition, for a shell formulation, an elemental thickness error vector is calculated as the difference between the thickness distribution of the deformed configuration and the measured in vivo configuration, $\mathbf {R_h}^k={\mathbf {h}}^k-{\mathbf {h}}^*$. The aim of the prestress algorithm is to drive these error vectors to zero by iteratively updating the guess for the unloaded configuration. To this end, the error vectors (with scaling factor, $\alpha$) are subtracted from the current guess for the unloaded configuration to obtain the unloaded configuration for the next iteration, $({\mathbf {X}}^{k+1},{\mathbf {H}}^{k+1})=({\mathbf {X}}^{k},{\mathbf {H}}^{k})-\alpha ({\mathbf {R}}^k,\mathbf {R_h}^k)$ . This iterative procedure is repeated until the maximum value of the error vectors falls below specified tolerances, i.e., the deformed configuration matches the medically imaged in vivo configuration.

In the original approach proposed by Sellier^37^, the scaling factor, $\alpha$, used to update the reference configuration was taken to be 1. Subsequent reports have revealed that this is not necessarily the best choice and the optimal value of scaling factor might be problem-dependent^41^. Furthermore, since this approach utilizes a fixed-point iteration method, it can lead to convergence issues for some problems. However, we did not encounter any such issues for the numerical examples reported in this work. We remark that the choice of scaling factor affects only the convergence rate and not the final result. In this work, we have not explored methods to potentially accelerate this rate of convergence and have employed a uniform scaling factor of 0.5.

## Results

### Verification with axisymmetric solution

First, we compare our numerical results against the well-known axisymmetric solution for a thick-walled cylindrical tube. Details of this solution are provided in Supplementary Appendix A online. This solution provides, in the most general case, the static mechanical response of a thick axisymmetric tube under combined bending, inflation, extension, and torsion^50,51^. Therefore, the solution of a thin wall tube (which is the object of verification in this study) is a special case of the more general solution.

**Figure 4:**
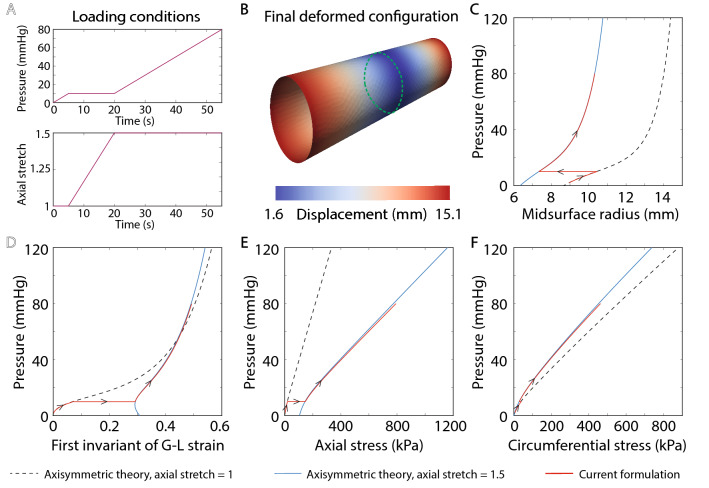
Verification of shell formulation for a cylindrical tube. (**A**) Shows the loading conditions, namely pressurization to 10 mmHg, followed by an extension to an axial stretch of 1.5 at constant pressure and, then a subsequent pressurization to 80 mmHg. (**B**) Shows the final deformed configuration at 80 mmHg. (**C**–**F**) Plot the evolution of midsurface radius, first invariant of Green-Lagrange strain tensor, axial stress, and circumferential stress, respectively.

For purposes of verification, we consider a distension-extension test where the vessel is represented as an incompressible cylindrical tube subjected to axial loading and pressurization. Under these loading conditions, the cylindrical tube deforms largely in the membrane mode, given appropriate boundary conditions at the ends (radial rollers). Consider a cylindrical tube with midsurface radius $r=8.6~\text {mm}$, axial length, $L=60~\text {mm}$, and wall thickness $t=0.43~\text {mm}$, resulting in a radius-to-thickness ratio of 20. For this verification, the arterial wall is assumed to be characterized by a four-fiber family model with material parameters listed in Supplementary Table S1 online. The tube is subjected to the loading conditions shown in Fig. 4A: pressurization to 10 mmHg, followed by an extension to an axial stretch of 1.5 at constant pressure and a subsequent pressurization to 80 mmHg. For the pressurization steps, the ends of the vessel are fixed in the axial direction. For the axial extension step, a non-zero displacement is prescribed on the ends to achieve the required axial stretch.

Figure 4B shows the final deformed configuration at 80 mmHg and an axial stretch of 1.5, obtained from the shell formulation. Fig. 4C–F compare the shell formulation results (red) against the axisymmetric theory predictions at axial stretch 1 and 1.5 at the location indicated by the green dotted circle in Fig. 4B. Consider four different metrics: midsurface radius, first invariant of Green-Lagrange strain tensor, and axial and circumferential Cauchy stress. The shell formulation results agree well with the axisymmetric theory for all the metrics considered, verifying our implementation. We remark that, in the absence of an imposed axial stretch (i.e., for axial stretch = 1), the pressurization of cylindrical segments is known to exhibit buckling instabilities^52,53^. Consequently, the axisymmetric solution for an axial stretch of 1 may not be physically attainable at high pressures. In this work, we only considered a mild pressurization to 10 mmHg for an axial stretch of 1 to avoid this instability and have highlighted the axisymmetric solution for higher inflation pressures via dotted green line to indicate this regime.

### Verification against literature data

**Figure 5:**
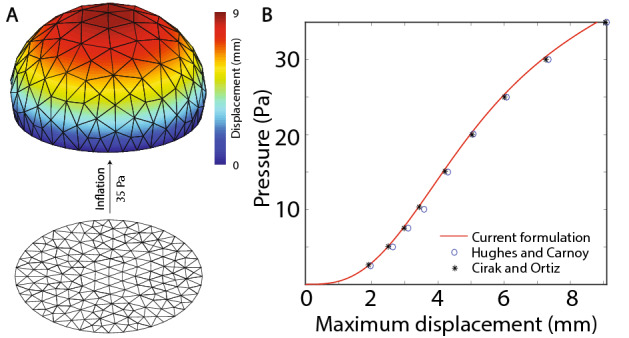
Verification of the current implementation against literature data. (**A**) Shows the initial and the deformed configuration of a simply supported circular plate inflated under uniform pressure. (**B**) Compares the pressure vs. maximum displacement obtained from our implementation against literature data.

Next, consider the shell formulation for combined membrane and bending deformation modes. A simply supported circular plate under uniform pressure has been previously used as a benchmark to assess the bending behavior of shell formulations^29,54^. The plate has a radius $r=7.5~\text {mm}$ and thickness $t=0.5~\text {mm}$, resulting in a radius-to-thickness ratio of 15. A Mooney-Rivlin material model, as described in “Constitutive models” section, was used with the material parameters $c_1=80~\text {Pa}$ and $c_2=20~\text {Pa}$. Figure 5A shows the initial $(P=0)$ and the deformed configuration of the plate $(P=35~\text {Pa})$. Figure 5B compares the pressure vs. maximum displacement obtained from our implementation against previously reported results. Specifically, we compare our results against two formulations: *(i)* subdivision elements reported by Cirak and Ortiz^29^ and, *(ii)* selectively-integrated 9 node axisymmetric solid elements reported by Hughes and Carnoy^54^. Our results agree well with these prior results, thereby demonstrating the ability of the current shell formulation to handle combined bending and membrane deformation modes with excellent accuracy.

### Prestress verification

Next, we verified our prestress methodology and evaluated the effect of inclusion of prestress in the analysis. Consider an idealized bifurcation model comprising a main artery and a side branch. The main vessel is a cylinder with radius $0.6~\text {mm}$ while the side branch is a cylinder with radius $0.2~\text {mm}$. The two branches meet at a right angle with a fillet radius of $0.2~\text {mm}$ at the blending region. The wall thickness is taken to be 10% of the local radius, resulting in wall thickness of $0.06~\text {mm}$ and $0.02~\text {mm}$ for the main vessel and the branch, respectively and a smooth variation of thickness in the blend region. We refer to this analytical configuration as the true unloaded configuration. Without a loss of generality, we assumed a Neo-Hookean material model with $c_1=0.16~\text {MPa}$. We employ *radially-free* boundary conditions for the ends such that nodes are free to move in the radial direction while fixed in the longitudinal direction, to enforce fixed axial extensions, and constrained circumferentially, to ensure axisymmetry.

To create the reference dataset for the verification of prestress algorithm, we first perform a standard forward analysis where we subject the true unloaded configuration to $P=80~{\text {mmHg}}$ and $P=120~{\text {mmHg}}$ to obtain true diastolic and true systolic configurations, respectively. Next, we pretend that the true diastolic configuration is obtained from medical imaging but the corresponding unloaded configuration is unknown. Hence, starting from the true diastolic configuration, the corresponding systolic configuration can be obtained via two possible analyses:

1. *With prestress*: Here, the true diastolic configuration is subjected to a prestress analysis to first obtain the corresponding unloaded configuration. Subsequently, this unloaded configuration is subjected to systolic pressure ($P=120~{\text {mmHg}}$) to obtain the systolic configuration.
2. *Without prestress*: Here, the true diastolic configuration is assumed to be the unloaded configuration and is directly subjected to systolic pressure ($P=120~{\text {mmHg}}$) to obtain the systolic configuration. This case mimics the frequently employed approach where subject-specific anatomies are simulated neglecting prestress^30,55,56^.

**Figure 6:**
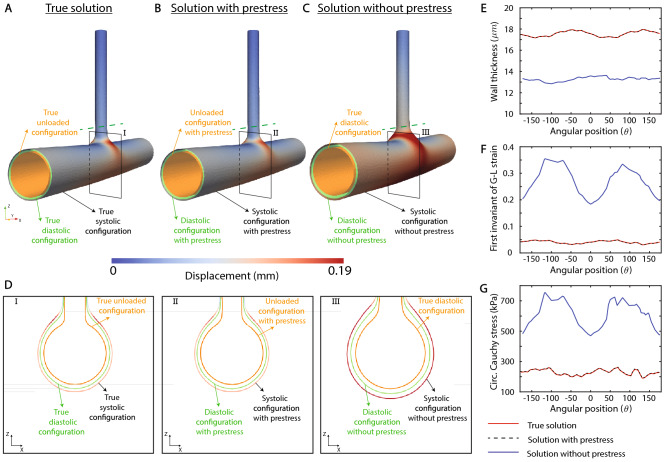
Prestress verification against forward solution. (**A**–**C**) show the different configurations pertaining to the true solution, solution with prestress, and solution without prestress, respectively. (**D**) shows the enlarged versions of these configurations along the planes I, II, and III indicated in (**A**–**C**). (**E**–**G**) show the comparison of circumferential variation of different metrics among true solution, solution with prestress, and solution without prestress at the side branch location indicated by dotted green line in (**A**–**C**).

Therefore, the analysis with prestress includes an intermediate step of identifying the unloaded configuration via the aforementioned prestress algorithm. Figure 6A–C show different configurations obtained via these analyses. Specifically, Fig. 6A shows the true unloaded, diastolic, and systolic configurations obtained via a standard forward analysis. Figure 6B,C show the corresponding configurations obtained from the analysis with and without prestress, respectively. Figure 6D shows the enlarged versions of these configurations along the planes I, II, and III indicated in Fig. 6A–C, respectively. Referring to the left and the middle column of Fig. 6D, we note that the predicted unloaded configuration with prestress is almost identical to the true unloaded configuration. Furthermore, the predicted systolic configuration with the inclusion of prestress agreed well with the true systolic configuration. In contrast, the predicted systolic configuration without the inclusion of prestress (right column of Fig. 6D) is much larger than the true systolic configuration (left column of Fig. 6D). Next, Fig. 6E–G show circumferential variations of different metrics in the predicted systolic configurations at an arbitrarily chosen location on the side branch, indicated by the dotted green lines in Fig. 6A–C. Specifically, we compare three different metrics in the predicted systolic configuration: wall thickness, first invariant of the Green-Lagrange strain tensor, and circumferential Cauchy stress. Again, results obtained with the inclusion of prestress are in excellent agreement with the true results while the results without the inclusion of prestress differ significantly from the true results for all the metrics.

### Application to subject-specific geometries

Next, we demonstrate the utility of our shell formulation and prestress algorithm for mouse-specific anatomies featuring multiple branches. These anatomies were obtained via non-invasive in vivo micro-CT and have been used in our previous work^12^. We employ an independently validated four-fiber family constitutive model to describe the behavior of the aortic wall. The regionally varying material parameters and distributions of wall thickness were obtained through in vitro biaxial tests (Supplementary Table S2 online). Further details about the experimental procedures can be found elsewhere^12^. We employ *radially-free* boundary conditions for the aortic wall ends, as described in “Prestress verification” section. In order to avoid unphysiological deformations and buckling of the arterial wall, it is crucial to include external tissue support boundary conditions to model the effect of surrounding tissue. Therefore, we incorporate the external tissue support conditions described in Eq. (74) with $k_s=10^5\, \text {g}/(\text {mm}^2\;\text {s}^2)$.

**Figure 7:**
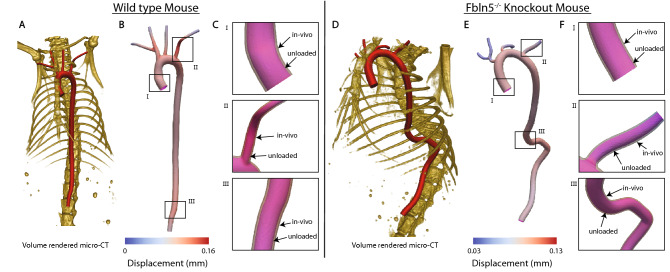
(**A**) Volume rendered geometric model of the aortic anatomy of a 20 weeks old wild type female mice. (**B**) Plots of the predicted unloaded configuration overlaid upon the medically imaged in vivo anatomy. (**C**) Enlarged version of unloaded and in vivo configurations at three different locations indicated in (**B**). (**D**) Volume rendered geometric model of the aortic anatomy of a $Fbln5^{-/-}$ knockout male mice. (**E**) Plots of the predicted unloaded configuration overlaid upon the medically imaged in vivo anatomy. (**F**) Enlarged version of unloaded and in vivo configurations at three different locations indicated in (**E**).

Figure 7A shows the volume rendered geometric model and the associated skeleton, obtained from the micro-CT data for a 20-week old wild-type mouse. Starting from this anatomy and the experimentally obtained wall properties (material parameters and thickness distribution), we employ our prestress algorithm to identify the corresponding unloaded geometry, which was not available in this anesthetized mouse. Figure 7B shows the predicted unloaded configuration overlaid on the medically imaged in vivo anatomy. Figure 7C shows enlarged views of the three different locations (I, II, III) indicated in Fig. 7B. It can be seen that the predicted unloaded configuration is contained within the in vivo configuration at all three locations. These results support the ability of our prestress algorihtm to handle complex, subject-specific anatomies with multiple branches.

To further test the robustness of our implementation, we repeat the same analysis on a $Fbln5^{-/-}$ knockout (KO) mouse. The regionally varying material parameters for the four-fiber constitutive model and the thickness distribution are listed in Supplementary Table S3 online. Figure 7D shows the corresponding volume rendered geometric model of the aortic anatomy and associated skeleton obtained from the micro-CT data. The KO anatomy exhibits a marked increase in tortuosity and therefore serves as a more stringent test to assess our implementation for complex, subject-specific anatomies. Figure 7E shows the predicted unloaded configuration overlaid on the medically imaged in vivo anatomy while Fig. 7F shows the corresponding enlarged version at three different locations indicated in Fig. 7E. As expected, the predicted unloaded configuration is smaller than the in vivo configuration. Note that the deformation of the aortic wall in the regions of vessel bifurcations is characterized by significant bending.

**Figure 8:**
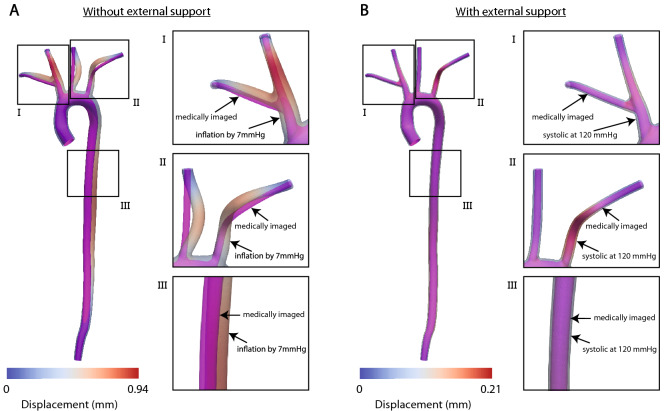
(**A**) Analysis without incorporation of external tissue support. The solution is characterized by unphysiological large deformations of the arterial wall leading to buckling in head branches and eventual failure of the simulation at $P=7~{\text {mmHg}}$. (**B**) Analysis with the incorporation of external tissue support. The systolic configuration at $P=120~{\text {mmHg}}$ is characterized by a physiological range of arterial wall deformations.

Figure 8 shows deformations of the arterial wall of a wild-type mouse without (panel A) or with (panel B) external tissue support. The solution without external tissue support exhibits unphysiological large deformations, leading to buckling in the head branches and eventual failure of the prestress analysis at $P=7~{\text {mmHg}}$. In contrast, the analysis with external tissue support exhibits no buckling and yields realistic deformations of arterial wall at $P=120~{\text {mmHg}}$.

Therefore, these results serve as a confirmation of the ability of our shell formulation to handle combined membrane and bending mode deformations in complex image-based multi-branched vascular models.

## Discussion and conclusions

We have implemented a geometrically and materially nonlinear rotation-free shell formulation capable of handling large deformations for vascular biomechanical applications. The formulation accounts for both membrane and bending behaviors with only displacement degrees of freedom by considering an element patch comprising the chosen element and the three adjacent elements. We performed static condensation of the thickness stretch using incompressibility and a plane stress condition; this provides a consistent method for incorporating arbitrary 3D constitutive models in our formulation. We also adapted a previously reported prestress algorithm to this shell formulation. Lastly, we incorporated the effect of surrounding tissue on vasculature by including external tissue support boundary conditions.

We verified the membrane behavior of the shell formulation by considering a distension–extension test on a cylindrical geometry and comparing our results against benchmark predictions. We further verified our shell formulation for combined membrane and bending modes by considering the deformation of a simply supported circular plate under uniform pressure, again with excellent agreement with prior findings by others.

Furthermore, we verified the prestress algorithm against standard forward calculations in an idealized bifurcation model. Our results indicate that given the in vivo pressure, anatomy, and associated mechanical properties (constitutive model, material parameters, and wall thickness), the prestress algorithm is able to accurately predict the unloaded configuration. This is an important demonstration given that both residual stresses (those existing in the traction-free configuration) and prestresses (those existing in vivo at diastolic pressure) are well known to influence significantly the calculated values of wall stress^50^, yet they are difficult to estimate directly via either experimental (e.g., opening angle tests and retraction measurements upon transection) or theoretical (e.g., growth and remodeling simulations, noting that residual and prestresses arise in part during vascular development) methods. Our results confirm that an analysis without prestress significantly overestimates the deformations of the arterial wall, thereby highlighting the need to include prestress in the analysis.

Lastly, we demonstrated the utility and robustness of our implementation by melding the shell formulation and prestress algorithm to identify the unloaded configuration of mouse-specific anatomies for wild-type and KO mice studied under anesthesia. These anatomies include multiple branches and exhibit significant bending around vessel bifurcations. Therefore, these cases serve as a test of the ability of our shell formulation to handle vascular deformations under complex membrane and bending modes. For these examples, we used an experimentally informed four-fiber constitutive material model to capture known nonlinearities and anisotropies, which vary by region. Our prestress algorithm predicted reasonable unloaded configurations for both anatomies, confirming the robustness of our implementation for application to complex, subject-specific anatomies.

The lack of rotational degrees of freedom in the presented nonlinear shell formulation makes it well-suited to serve as the structural model for a strongly coupled, monolithic, computationally efficient, nonlinear fluid-structure interaction framework via an adaption of the CMM to large displacements. Although prior simulations, including our own^12,13^, have proven useful in studying fluid–solid interactions in many regions of the vasculature, the present formulation promises to be better suited to study ventricular-vascular interactions, particularly since the ascending aorta experiences dramatic finite distension, extension, and bending during the cardiac cycle. The presented shell formulation is also suitable for including growth and remodeling by using a constrained mixture theory (CMT)^57^. Including CMT in future fluid-solid simulations will extend our prior work^58^ to subject-specific geometries and large displacements and thereby improve the understanding of the interplay between altered hemodynamics and vascular adaptation. Since the current shell formulation is based only on displacement degrees of freedom, it also enables standard integration with the methodologies proposed in literature to perform acute stress analysis on stents^59^, to investigate vascular adaptation following the stent implantation^60^, and indeed to consider a host of clinical interventions for patient-specific geometries.

## Supplementary information

Supplementary Information.

## Data Availability

All data generated or analysed during this study are included in this published article (and its Supplementary Information files).
