# Supplementary material for: A nonlinear rotation-free shell formulation with prestressing for vascular biomechanics
Source: Sci Rep. 2020 Oct 16;10:17528. doi: 10.1038/s41598-020-74277-5 (PMC7567841; doi:10.1038/s41598-020-74277-5)
Supplement: Supplementary file 1 — Supplementary Information. [file 41598_2020_74277_MOESM1_ESM.pdf]

Supplementary material

A Nonlinear Rotation-free Shell Formulation with Prestressing for Vascular Biomechanics

Nitesh Nama[1,*], Miquel Aguirre[2], Jay D. Humphrey[3], and C. Alberto Figueroa[1,4]

[1]Department of Surgery, University of Michigan, Ann Arbor, MI, USA
[2]Mines Saint-Étienne, Univ Lyon, Univ Jean Monnet, INSERM, U 1059 Sainbiose, Centre CIS, F - 42023 Saint-Étienne, France
[3]Department of Biomedical Engineering, Yale University, New Haven, CT, USA
[4]Department of Biomedical Engineering, University of Michigan, Ann Arbor, MI, USA
[*]Email: nnama@umich.edu

A Axisymmetric theory

In order to validate the proposed framework, it is useful to recall the well-known thick wall cylindrical tube solution. This solution provides, in the most general case, the static mechanical response of a thick axisymmetric tube under combined inflation, extension, and torsion [1, 2]. Here, we focus on inflation and extension. The vessel is assumed to be an incompressible thick-walled cylindrical tube that undergoes a finite deformation from an undeformed configuration Ω_0 to the current configuration Ω. For the comparison purposes of the example in section 5.1, no residual stress will be considered. Considering all of the above, configuration Ω_0 is given in terms of cylindrical coordinates (R,Θ,Z) as

$$R_i \leq R \leq R_o, \quad 0 \leq \Theta \leq 2\pi, \quad 0 \leq Z \leq L, \tag{S1}$$

with R_i, R_o and L the initial inner and outer radii and initial length of the tube. Similarly, the cylindrical coordinates (r,θ,z) define the current configuration Ω as

$$r_i \leq r \leq r_o, \quad 0 \leq \theta \leq 2\pi, \quad 0 \leq z \leq l, \tag{S2}$$

with r_i, r_o and l the current inner and outer radii and current length of the tube. In the absence of torsion and bending, the current cylindrical coordinates can be written in terms of the initial coordinates as

$$r = \sqrt{\frac{R^2 - R_i^2}{\lambda_z} + r_i^2}, \quad \theta = \Theta, \quad z = \lambda_z Z, \tag{S3}$$

with λ_z the axial stretch. Additionally, the radial and circumferential stretches, λ_r, λ_θ are defined as

$$\lambda_r = \frac{\partial r}{\partial R} = \frac{R}{r\lambda_z}, \quad \lambda_\theta = \frac{r}{R}\frac{\partial \theta}{\partial \Theta}. \tag{S4}$$

The three stretches are related through the incompressibility condition as

$$\lambda_r \lambda_\theta \lambda_z = 1, \tag{S5}$$

and allow defining the Green Lagrange strain tensor components as

$$E_{\Theta\Theta} = \frac{1}{2}\left(\lambda_\theta^2 - 1\right), \quad E_{RR} = \frac{1}{2}\left(\lambda_r^2 - 1\right), \quad E_{ZZ} = \frac{1}{2}\left(\lambda_z^2 - 1\right). \tag{S6}$$

The equilibrium equation, in the absence of body forces, can be written in cylindrical coordinates as

$$\frac{d\sigma_{rr}}{dr} + \frac{(\sigma_{rr} - \sigma_{\theta\theta})}{r} = 0. \tag{S7}$$

Taking into account that the tube is loaded by an internal pressure $p_i = -\sigma_{rr}(r = r_i)$ and that no load is applied on the outer wall, i.e. $\sigma_{rr}(r = r_o) = 0$, the above equation can be integrated across the thickness to yield

$$p_i = \int_{r_i}^{r_o} (\sigma_{\theta\theta} - \sigma_{rr}) \frac{dr}{r}, \tag{S8}$$

with the stress components $\sigma_{\theta\theta}$, σ_{rr} obtained via some constitutive relationship, i.e.

$$\sigma_{\theta\theta} = \sigma_{\theta\theta}(\lambda_\theta, \lambda_z, \lambda_r), \tag{S9}$$

$$\sigma_{rr} = \sigma_{rr}(\lambda_\theta, \lambda_z, \lambda_r). \tag{S10}$$

Equation (S8), combined with the constitutive relationships in equations (S9) and (S10), yields a nonlinear equation that allows computation of the current internal radius r_i as a function of the loading pressure p_i and axial stretch λ_z.

The constitutive relationship in cylindrical coordinates for an incompressible material can be derived using a Lagrange multiplier, as described in Section 2.4 or by enforcing incompressibility directly into the strain energy function via equation (S5). Following the approach described in [1] , a general 3D SEF ψ_{el} can be written in terms of only two principal strains, i.e.

$$\bar{\psi}_{el}(E_{\Theta\Theta}, E_{ZZ}) = \psi_{el}(E_{RR}, E_{\Theta\Theta}, E_{ZZ}), \tag{S11}$$

where $\bar{\psi}_{el}$ is the equivalent of the strain energy function that only depends on $E_{\Theta\Theta}$, E_{RR}. Using $\bar{\psi}_{el}$ and assuming that the principal components of stress and strain coincide, the following is obtained (see [1] for details)

$$\sigma_{\theta\theta} - \sigma_{rr} = (1+2E_{\Theta\Theta})\frac{\partial \bar{\psi}_{el}}{\partial E_{\Theta\Theta}}, \tag{S12}$$

$$\sigma_{zz} - \sigma_{rr} = (1+2E_{ZZ})\frac{\partial \bar{\psi}_{el}}{\partial E_{ZZ}}, \tag{S13}$$

which can be used in the nonlinear equation (S8). The partial derivative of $\bar{\psi}_{el}$ respect to $E_{\Theta\Theta}$ can be found using the chain rule and equations (S5) and (S6) for an appropriate strain energy function.

B Variation of membrane strain and curvature matrices

B.1 Variation of membrane strain

Using the assumed strain approach proposed by Oñate and Flores [3], the membrane strain field is considered as a linear interpolation of the values at the three edge midpoints of the master element. Consequently, for a reduced integration strategy with a single in-plane quadrature point per element, the variation of the Green-Lagrange membrane strain matrix can be expressed as

$$\begin{aligned}
\delta \begin{bmatrix} E^{\mathrm{m}}_{11} \\ E^{\mathrm{m}}_{22} \\ 2E^{\mathrm{m}}_{12} \end{bmatrix} &= \begin{bmatrix} \varphi_{,1}\cdot\delta\varphi_{,1} \\ \varphi_{,2}\cdot\delta\varphi_{,2} \\ \varphi_{,1}\cdot\delta\varphi_{,2}+\varphi_{,2}\cdot\delta\varphi_{,1} \end{bmatrix} \\
&= \sum_{I=1}^{3} L^I \begin{bmatrix} \varphi^I_{,1}\cdot\delta\varphi^I_{,1} \\ \varphi^I_{,2}\cdot\delta\varphi^I_{,2} \\ \varphi^I_{,1}\cdot\delta\varphi^I_{,2}+\varphi^I_{,2}\cdot\delta\varphi^I_{,1} \end{bmatrix} \\
&= \sum_{I=1}^{3}\sum_{a=1}^{6} L^I \begin{bmatrix} \left(\varphi^I_{,1}N^I_{a,1}\right)^T \\ \left(\varphi^I_{,2}N^I_{a,2}\right)^T \\ \left(\varphi^I_{,1}N^I_{a,2}+\varphi^I_{,2}N^I_{a,1}\right)^T \end{bmatrix} \delta\varphi_a \\
&= \mathbf{B}^{\mathrm{p}}_{\mathrm{m}}\delta\varphi^{\mathrm{p}},
\end{aligned} \tag{S14}$$

where $\delta\varphi^{\mathrm{p}}$ denotes the 18×1 vector gathering the variation of the positions of the 6 nodes belonging to the element patch

$$\delta\varphi^{\mathrm{p}} = [\delta\varphi_1^T, \delta\varphi_2^T, \delta\varphi_3^T, \delta\varphi_4^T, \delta\varphi_5^T, \delta\varphi_6^T]^T, \tag{S15}$$

and $\mathbf{B}^{\mathrm{p}}_{\mathrm{m}}$ denotes the 3×18 matrix given as

$$\mathbf{B}^{\mathrm{p}}_{\mathrm{m}} = [\mathbf{B}^1_{\mathrm{m}}, \mathbf{B}^2_{\mathrm{m}}, \mathbf{B}^3_{\mathrm{m}}, \mathbf{B}^4_{\mathrm{m}}, \mathbf{B}^5_{\mathrm{m}}, \mathbf{B}^6_{\mathrm{m}}], \tag{S16}$$

with

$$\mathbf{B}^a_{\mathrm{m}} = \frac{1}{3}\begin{bmatrix} \left(\varphi^1_{,1}N^1_{a,1}+\varphi^2_{,1}N^2_{a,1}+\varphi^3_{,1}N^3_{a,1}\right)^T \\ \left(\varphi^1_{,2}N^1_{a,2}+\varphi^2_{,2}N^2_{a,2}+\varphi^3_{,2}N^3_{a,2}\right)^T \\ \left(\varphi^1_{,1}N^1_{a,2}+\varphi^1_{,2}N^1_{a,1}+\varphi^2_{,1}N^2_{a,2}+\varphi^2_{,2}N^2_{a,1}+\varphi^3_{,1}N^3_{a,2}+\varphi^3_{,2}N^3_{a,1}\right)^T \end{bmatrix}. \tag{S17}$$

B.2 Variation of curvature

The variation of curvature is given as

$$\delta\chi_{\alpha\beta} = \delta\kappa_{\alpha\beta} = \delta\mathbf{h}_{\alpha\beta}\cdot\mathbf{a}_3 + \mathbf{h}_{\alpha\beta}\cdot\delta\mathbf{a}_3. \tag{S18}$$

Therefore, the first term on RHS of equation (S18) can be written as

$$\delta\mathbf{h}_{\alpha\beta}\cdot\mathbf{a}_3 = \sum_{I=1}^{3}\sum_{a=1}^{6}\left[L^I_{,\alpha}N^I_{a,\beta}+L^I_{,\beta}N^I_{a,\alpha}\right](\mathbf{a}_3\cdot\delta\varphi_a). \tag{S19}$$

Accordingly, the second term on RHS of equation (S18)

$$\mathbf{h}_{\alpha\beta}\cdot\delta\mathbf{a}_3 = \left[(-\mathbf{a}_3\cdot\delta\varphi^M_{,1})\breve{\mathbf{a}}_{,1} + (-\mathbf{a}_3\cdot\delta\varphi^M_{,2})\breve{\mathbf{a}}_{,2}\right]\cdot\mathbf{h}_{\alpha\beta}$$
$$= \left[-\sum_{I=1}^{3}\left[L^I_{,1}\breve{\mathbf{a}}_{,1} + L^I_{,2}\breve{\mathbf{a}}_{,2}\right](\mathbf{a}_3\cdot\delta\varphi_I)\right]\cdot\mathbf{h}_{\alpha\beta}$$
$$= -\sum_{I=1}^{3}\left[L^I_{,1}(\breve{\mathbf{a}}_{,1}\cdot\mathbf{h}_{\alpha\beta}) + L^I_{,2}(\breve{\mathbf{a}}_{,2}\cdot\mathbf{h}_{\alpha\beta})\right](\mathbf{a}_3\cdot\delta\varphi_I), \tag{S20}$$

where $\breve{\mathbf{a}}_\alpha$ are contravariant basis vectors in the master element defined using the linear interpolation and obtained as

$$\breve{\mathbf{a}}_1 = \frac{1}{\sqrt{\det(a^M_{\alpha\beta})}}\varphi^M_{,2}\times\mathbf{a}_3, \tag{S21}$$

$$\breve{\mathbf{a}}_2 = -\frac{1}{\sqrt{\det(a^M_{\alpha\beta})}}\varphi^M_{,1}\times\mathbf{a}_3, \tag{S22}$$

with $a^M_{\alpha\beta} = \varphi^M_{,\alpha}\cdot\varphi^M_{,\beta}$. Combining these expressions, the variation of curvature is given as

$$\delta\chi_{\alpha\beta} = \sum_{I=1}^{3}\sum_{a=1}^{6}\left[L^I_{,\alpha}N^I_{a,\beta} + L^I_{,\beta}N^I_{a,\alpha}\right](\mathbf{a}_3\cdot\delta\varphi_a)$$
$$-\sum_{I=1}^{3}\left[L^I_{,1}(\breve{\mathbf{a}}_{,1}\cdot\mathbf{h}_{\alpha\beta}) + L^I_{,2}(\breve{\mathbf{a}}_{,2}\cdot\mathbf{h}_{\alpha\beta})\right](\mathbf{a}_3\cdot\delta\varphi_I). \tag{S23}$$

C Time integration and linearization

We employ a Generalized-α time integration scheme [4] to solve the linearized equation of linear momentum balance. To this end, we consider variations of δW^{kin}, δW^{int}, and δW^{ext} described in section 3 with respect to the discretized displacement vector to obtain the vectors of kinetic, internal, and external nodal forces as

$$\mathbf{F}^{\text{kin}}_{\text{a}} = \int_A \rho_0 H\ddot{\mathbf{u}}\cdot\frac{\partial\mathbf{u}}{\partial\mathbf{u}_a}\,dA \tag{S24}$$

$$\mathbf{F}^{\text{int}}_{\text{a}} = \int_A \left[\mathbf{N}:\frac{\partial\mathbf{E}^{\text{m}}}{\partial\mathbf{u}_a} + \mathbf{M}:\frac{\partial\chi}{\partial\mathbf{u}_a}\right]dA \tag{S25}$$

$$\mathbf{F}^{\text{ext}}_{\text{a}} = \int_A \mathbf{f}\cdot\frac{\partial\mathbf{u}}{\partial\mathbf{u}_a}\,dA \tag{S26}$$

where the partial derivatives of $\mathbf{E}^{\text{m}}$ and χ are obtained, respectively, from equations (S14) and (S23). Using the results above, and upon assembly, the discrete nonlinear equation for linear momentum balance can be written as

$$\mathbf{R}(\ddot{\mathbf{U}},\dot{\mathbf{U}},\mathbf{U},t) = \mathbf{F}^{\text{ext}} - \mathbf{F}^{\text{kin}} - \mathbf{F}^{\text{int}} = \mathbf{0}, \tag{S27}$$

where $\mathbf{R}$ is the assembled residual vector, $\mathbf{F}^{\text{ext}}$, $\mathbf{F}^{\text{kin}}$ and $\mathbf{F}^{\text{int}}$ are, respectively, the assembled external, kinetic and internal force vectors and $\ddot{\mathbf{U}}$, $\dot{\mathbf{U}}$, $\mathbf{U}$ are, respectively, the global vector of nodal accelerations, velocities and displacements. The nonlinear, transient system of equation (S27) is solved using the generalized alpha method, where the residual is evaluated as

$$\mathbf{R}(\ddot{\mathbf{U}}_{n+\alpha_M},\dot{\mathbf{U}}_{n+\alpha_F},\mathbf{U}_{n+\alpha_F}) = \mathbf{0}, \tag{S28}$$

with α_M, α_F the time integrator parameters (see reference [4] for details). The above system of equations is solved using Newton-Raphson technique, in the form

$$\tilde{\mathbf{K}}\Delta\ddot{\mathbf{U}} = -\mathbf{R}, \tag{S29}$$

with

$$\tilde{\mathbf{K}} = \alpha_M\mathbf{M} + \alpha_F(\Delta t\gamma)\mathbf{C} + \alpha_F\left(\Delta t^2\beta\right)\mathbf{K}, \tag{S30}$$

with Δt the time step size, γ, β the Newmark time integration parameters, $\mathbf{M}$ the mass matrix, $\mathbf{C}$ the damping matrix and $\mathbf{K}$ the stiffness matrix. We will not provide details in the derivation of the mass matrix (standard in Finite Element) nor in the derivation of the damping matrix (the contribution of which comes from the external tissue support). Regarding the stiffness matrix, the elemental contribution (before assembly) for a couple of nodes a, b is given as

$$\mathbf{K}_{\text{ab}} = \mathbf{K}^{\text{ext}}_{\text{ab}} - \mathbf{K}^{\text{int}}_{\text{ab}}, \tag{S31}$$

with

$$\mathbf{K}^{\text{int}}_{\text{ab}} = \int_A \left[\frac{\partial \mathbf{N}}{\partial \mathbf{u}_b} : \frac{\partial \mathbf{E}^{\text{m}}}{\partial \mathbf{u}_a} + \mathbf{N} : \frac{\partial^2 \mathbf{E}^{\text{m}}}{\partial \mathbf{u}_a \partial \mathbf{u}_b} + \frac{\partial \mathbf{M}}{\partial u_b} : \frac{\partial \chi}{\partial \mathbf{u}_a} + \mathbf{M} : \frac{\partial^2 \chi}{\partial \mathbf{u}_a \partial \mathbf{u}_b} \right] dA, \tag{S32}$$

$$\mathbf{K}^{\text{ext}}_{\text{ab}} = \int_A \frac{\partial \mathbf{f}}{\partial \mathbf{u}_b} \cdot \frac{\partial \mathbf{u}}{\partial \mathbf{u}_a} \, dA. \tag{S33}$$

as a 3×3 matrix:

$$\bar{\mathscr{C}} = \begin{bmatrix} \mathscr{C}^{1111} & \mathscr{C}^{1122} & \mathscr{C}^{1112} \\ \mathscr{C}^{1122} & \mathscr{C}^{2222} & \mathscr{C}^{2212} \\ \mathscr{C}^{1112} & \mathscr{C}^{2212} & \mathscr{C}^{1212} \end{bmatrix}, \tag{S34}$$

and introduce the following thickness integrations of this matrix

$$\bar{\mathscr{C}}^{(0)} = \int_{-H/2}^{H/2} \bar{\mathscr{C}} \, d\xi_3, \tag{S35}$$

$$\bar{\mathscr{C}}^{(1)} = \int_{-H/2}^{H/2} \xi_3 \bar{\mathscr{C}} \, d\xi_3, \tag{S36}$$

$$\bar{\mathscr{C}}^{(2)} = \int_{-H/2}^{H/2} \xi_3^2 \bar{\mathscr{C}} \, d\xi_3. \tag{S37}$$

Using Voigt notation to express the components of various tensors as

$$\bar{\mathbf{N}} = \begin{bmatrix} N^{11} \\ N^{22} \\ N^{12} \end{bmatrix}, \qquad \bar{\mathbf{M}} = \begin{bmatrix} M^{11} \\ M^{22} \\ M^{12} \end{bmatrix}, \qquad \bar{\mathbf{E}}^{\text{m}} = \begin{bmatrix} E^{\text{m}}_{11} \\ E^{\text{m}}_{22} \\ 2E^{\text{m}}_{12} \end{bmatrix}, \qquad \bar{\chi} = \begin{bmatrix} \chi_{11} \\ \chi_{22} \\ 2\chi_{12} \end{bmatrix}, \tag{S38}$$

the above expressions allow us to write the internal component of tangential stiffness matrix as

$$\begin{aligned} \mathbf{K}^{\text{int}}_{\text{ab}} = \int_A \Bigg[& \left(\bar{\mathscr{C}}^{(0)} \frac{\partial \bar{\mathbf{E}}^{\text{m}}}{\partial \mathbf{u}_b} + \bar{\mathscr{C}}^{(1)} \frac{\partial \bar{\chi}}{\partial \mathbf{u}_b} \right)^T \frac{\partial \bar{\mathbf{E}}^{\text{m}}}{\partial \mathbf{u}_a} + \left(\bar{\mathscr{C}}^{(1)} \frac{\partial \bar{\mathbf{E}}^{\text{m}}}{\partial \mathbf{u}_b} + \bar{\mathscr{C}}^{(2)} \frac{\partial \bar{\chi}}{\partial \mathbf{u}_b} \right)^T \frac{\partial \bar{\chi}}{\partial \mathbf{u}_a} \\ & + \bar{\mathbf{N}}^T \frac{\partial^2 \bar{\mathbf{E}}^{\text{m}}}{\partial \mathbf{u}_a \partial u_b} + \bar{\mathbf{M}}^T \frac{\partial^2 \bar{\chi}}{\partial \mathbf{u}_a \partial \mathbf{u}_b} \Bigg] dA. \end{aligned} \tag{S39}$$

Here, the terms in the first row represent the material component of the stiffness matrix while the terms in the second row represent the geometric component of the stiffness matrix.

D Material parameters for the four-fiber family constitutive model

c (kPa)	c_1^1 (kPa)	c_2^1	c_1^2 (kPa)	c_2^2	$c_1^{3,4}$ (kPa)	$c_2^{3,4}$	α_0(deg)
37.20	4.68e-6	2.97e-7	2.9e-5	1.68e-5	28.16	3.48	43.88

Table S1. Material parameters for validation with axisymmetric theory. These parameters correspond to the material parameters for a human descending aorta reported in reference [5].

Wall segment	c (kPa)	c_1^1 (kPa)	c_2^1	c_1^2 (kPa)	c_2^2	$c_1^{3,4}$ (kPa)	$c_2^{3,4}$	α_0(deg)	h (μm)
ATA	20.99	18.50	0.026	18.91	0.062	4.90	0.410	40.7	53.4
DTA	12.74	25.10	0.170	14.36	0.062	0.43	1.935	23.3	47.0
SAA	20.83	23.34	0.074	13.11	0.093	0.67	1.189	30.6	43.0
IAA	9.31	12.12	0.116	13.25	0.120	0.55	0.899	33.0	35.0
CCA	5.05	20.59	0.054	7.66	0.044	0.02	1.522	34.1	28.0

Table S2. Material parameters of the four-fiber constitutive model for wild type mouse for different aortic wall segments denoted by ATA- Ascending Thoracic Aorta, DTA-Descending Thoracic Aorta, SAA-Suprarenal Abdominal Aorta, IAA-Infrarenal Abdominal Aorta, and CCA-Common Carotid Arteries.

Wall segment	c (kPa)	c_1^1 (kPa)	c_2^1	c_1^2 (kPa)	c_2^2	$c_1^{3,4}$ (kPa)	$c_2^{3,4}$	α_0(deg)	h (μm)
ATA	18.26	11.86	0.349	9.03	0.787	1.62	1.63	37.0	71.0
DTA	13.67	7.45	2.212	12.79	0.418	0.58	3.87	28.1	59.5
SAA	27.18	11.07	3.911	8.36	0.645	2.24	4.85	25.8	51.2
IAA	16.48	7.47	1.847	9.06	0.480	0.30	4.05	26.8	56.0
CCA	16.30	9.23	4.051	7.40	0.726	0.38	5.33	26.8	39.4

Table S3. Material parameters of the four-fiber constitutive model for $Fbln5^{-/-}$ knockout (KO) mouse for different aortic wall segments denoted by ATA- Ascending Thoracic Aorta, DTA-Descending Thoracic Aorta, SAA-Suprarenal Abdominal Aorta, IAA-Infrarenal Abdominal Aorta, and CCA-Common Carotid Arteries.

References

1. Humphrey, J. D. *Cardiovascular solid mechanics: cells, tissues, and organs* (Springer Science & Business Media, 2013).

2. Holzapfel, G. A., Gasser, T. C. & Ogden, R. W. A new constitutive framework for arterial wall mechanics and a comparative study of material models. *J. elasticity physical science solids* **61**, 1–48 (2000).

3. Oñate, E. & Flores, F. G. Advances in the formulation of the rotation-free basic shell triangle. *Comput. methods applied mechanics engineering* **194**, 2406–2443 (2005).

4. Chung, J. & Hulbert, G. A time integration algorithm for structural dynamics with improved numerical dissipation: the generalized-α method. (1993).

5. Roccabianca, S., Figueroa, C., Tellides, G. & Humphrey, J. Quantification of regional differences in aortic stiffness in the aging human. *J. mechanical behavior biomedical materials* **29**, 618–634 (2014).
